# Polarization transformation and destructive interference on subwavelength magnetic domains in magneto-plasmonic systems

**DOI:** 10.1038/s41598-022-17971-w

**Published:** 2022-08-16

**Authors:** Haruki Yamane, Satoshi Yanase, Takashi Hasegawa, Masanobu Kobayashi, Yukiko Yasukawa

**Affiliations:** 1 Akita Industrial Technology Center, 4-21 Sanuki, Araya, Akita, 010-1623 Japan; 2 Akita University, 1-1 Tegata, Gakuen-machi, Akita, 010-8502 Japan; 3 Chiba Institute of Technology, 2-17-1 Tsudanuma, Narashino, Chiba 275-0016 Japan

**Keywords:** Applied physics, Optical physics, Nanoscale materials, Optical materials and structures, Condensed-matter physics, Materials for optics, Nanoscale materials

## Abstract

We demonstrate magneto-optical (MO) polarization transformation due to surface plasmons in CoPt perpendicular magnetic films in the polar Kerr geometry. An extraordinary Kerr rotation angle (*θ*_K_ =  ± 88.9°) that almost reaches the upper limit of polarization is produced in the attenuated total reflection (Kretschmann) configuration. *P*-polarized incident radiation is almost transformed upon reflection to *s*-polarized radiation, which may be out of phase depending on whether the magnetization of CoPt is up or down. Moreover, the reflected intensity may be drastically modulated by applying an external magnetic field. The reflectivity goes almost to zero in the demagnetized state and increases with increasing external magnetic field. This drastic optical response is attributed to the MO destructive interference produced by the subwavelength magnetic domain structure.

## Introduction

Magneto-plasmonics, which combines magnetic and plasmonic functionalities, has been intensively investigated, theoretically and experimentally. The strong electromagnetic field induced by surface-plasmon resonances (SPRs) significantly enhances and modulates the magneto-optical (MO) Kerr and Faraday effects^1–8^. Large MO activities are desirable in practical applications such as information storage systems, telecommunications, and chemical and biological sensors. Magneto-plasmonic systems in which amplitude, phase, polarization, and chirality are substantially tunable by applying an external magnetic field might lead to new active photonic devices^9–14^.

Surface plasmons in ferromagnetic materials can exist in a wide variety of metallic structures, such as multilayers^1–3^, nanoparticles^4,11,15^, and patterned arrays^5,6,16^. They are classified into two main categories: surface plasmon polaritons (SPPs) that propagate on planar interfaces and localized surface plasmons in subwavelength structures. Stacked-layer structures that consist of noble metals (Au and Ag) and magnetic transition metals (e.g., Co, Fe, and Ni) constitute particularly interesting propagating-SPP systems. The Kretschmann (or attenuated total reflection, ATR) configuration^17^, which is commonly used for exciting SPPs, is useful for optical devices because of its simple fabrication and assembly.

From the magnetic point of view, MO phenomena are classified into in-plane (transversal or longitudinal) and perpendicular (polar) magnetic geometries. For propagating SPPs (nonlocalized systems), the transverse Kerr excitation was used in most previous studies. A theoretical study suggests that high-quality surface plasmons are the most effective in enhancing the MO effect in the transverse geometry^18^. Large transverse Kerr activities have been demonstrated in in-plane magnetic films comprising Au/Co/Au trilayers^2,3^. A small magnetic saturation field in common in-plane magnetic materials is also useful for sensor equipment that exploits magnetic synchronizing techniques. Finally, in-plane magnetic SPR elements substantially improve the signal-to-noise ratio, sensitivity, and detection limit of chemical and biological sensors^19–21^.

Besides the MO activities, perpendicular magnetics is one of the most significant technologies in next-generation magnetic and spintronic applications for promoting thermal stability and device miniaturization^22,23^. Perpendicular magnetic materials are a prerequisite for high-density magnetic and MO recording media, high-resolution MO spatial light modulators, and large-capacity magnetic random-access memories. Co‒Pt films (alloys, multilayers, and nanoparticles) are particularly interesting candidates for these applications because of their large perpendicular magnetic anisotropy and high chemical and thermal stability. Hexagonal close-packed (hcp) Co_80_Pt_20_-based films are used as high-density perpendicular magnetic recording media in commercial products, and *L*1_0_–Co_50_Pt_50_ composite films and Co/Pt multilayers have been investigated for use in next-generation high-density recording media and spintronic devices^22,24–26^. Furthermore, their relatively large MO activity in the short (blue) wavelengths and control of the polarization in the polar MO geometry are potentially useful aspects for MO devices. Several theoretical and experimental studies have also shown that the SPRs significantly enhance polar Kerr activities^1,2,27^. We recently reported that SPR elements comprising perpendicular magnetic Co_80_Pt_20_/ZnO/Ag trilayers produce a giant polarization angle with narrow linewidth under polar Kerr excitation^28^. A new type of chemical sensor for detecting hydrogen gas was also demonstrated in the CoPt trilayers with an ideal square-shaped magnetic hysteresis loop.

In most previous studies on nonlocalized magneto-plasmonics, noble/ferromagnetic metal stacked structures serve as MO‒SPR elements in the ATR configuration because the noble metals (Ag and Au) produce high-quality SPPs. Magnetic transition metals generally produce a strong absorption loss, resulting in overdamped SPR^2,29^. A lot of theoretical and experimental studies have investigated optimized layered structures in which the plasmon excitation and MO response are mainly produced in the noble metal and ferromagnetic layers, respectively. By contrast, MO‒SPR elements consisting of pure ferromagnetic materials are not special in localized magneto-plasmonic systems or magneto-plasmonic crystals. For example, Ni, Fe, and Co magnetic nanoholes strongly enhance Kerr effects^5,30–32^. In the present study, we investigate how propagating SPPs affect the polar Kerr activities of single ferromagnetic metals in nonlocalized plasmonics. Herein, we investigated optical and MO responses in SPR elements, which primarily comprise hcp Co_80_Pt_20_ magnetic single layers in the polar Kerr geometry and in which the CoPt layers serve both for SPP excitation and to produce MO effects.

## Results

### Polar Kerr activities

**Figure 1:**
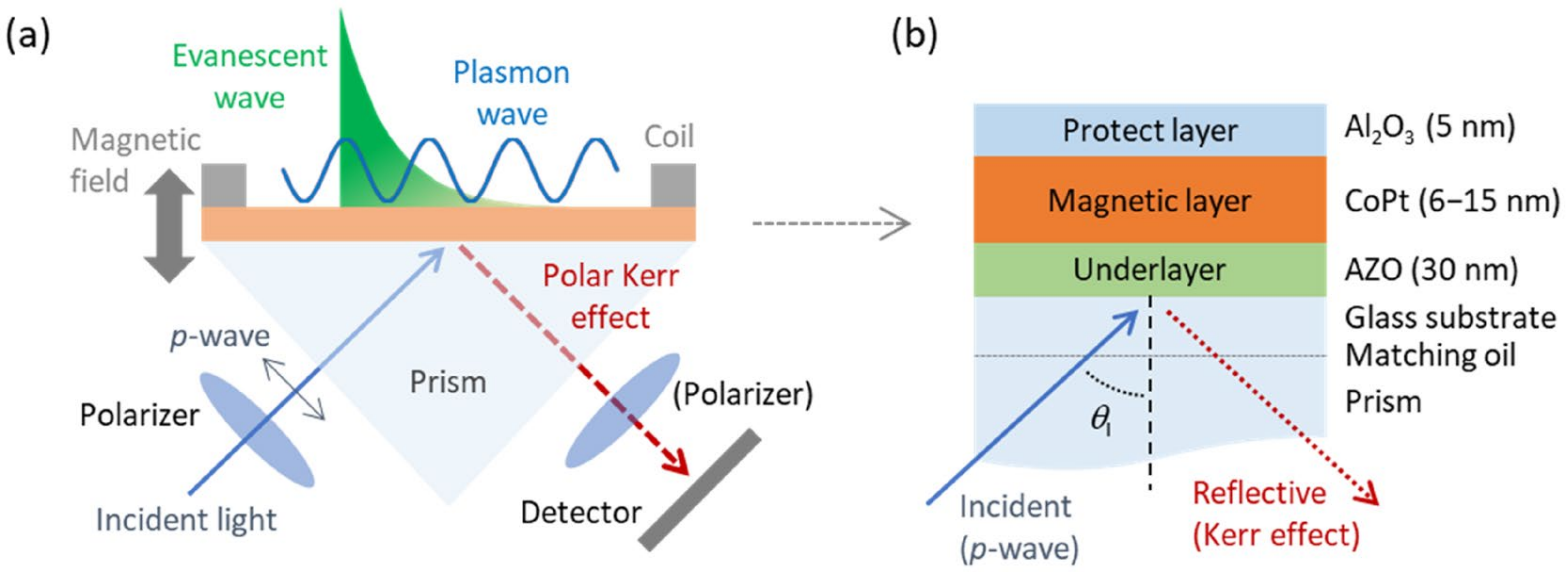
Schematics of (**a**) measurement setup for polar Kerr excitation geometry and (**b**) sample structure of MO–SPR elements. SPPs are excited in the Kretschmann (or attenuated total reflection) configuration with prism coupling, and an external magnetic field is applied along the normal to the sample surface. MO activities and reflected intensity are measured in SPR geometry with and without analyzing polarizer, respectively.

Figure 1a,b shows the schematics of a measurement setup for polar Kerr excitation and MO‒SPR element, respectively. The optical and MO responses of the samples were measured in the Kretschmann (or ATR) configuration using 658-nm *p*-polarized laser irradiation. The MO‒SPR element comprises a CoPt perpendicular magnetic thin film with a 5-nm-thick Al_2_O_3_ protective layer and a 30-nm-thick Al-doped ZnO (AZO) underlayer. The Al_2_O_3_/CoPt/AZO trilayers deposited onto glass substrates were optically coupled with a prism using an index-matching fluid. SPP excitation was controlled by tuning the incident angle *θ*_I_ of the laser irradiation and varying the thickness of the CoPt layer from 6 to 15 nm. An external magnetic field was applied along the surface normal of the samples to produce the geometry appropriate for the polar Kerr configuration. Reflected radiation was detected by a photomultiplier tube, and the MO activities (Kerr rotation angle *θ*_K_ and ellipticity *η*_K_) were determined using a polarizer. The reflected intensity *I*_R_ from the samples were obtained by the SPR measurement system without the analyzing polarizer. Sample preparation and experimental setup are described in the “Methods” section.

**Figure 2:**
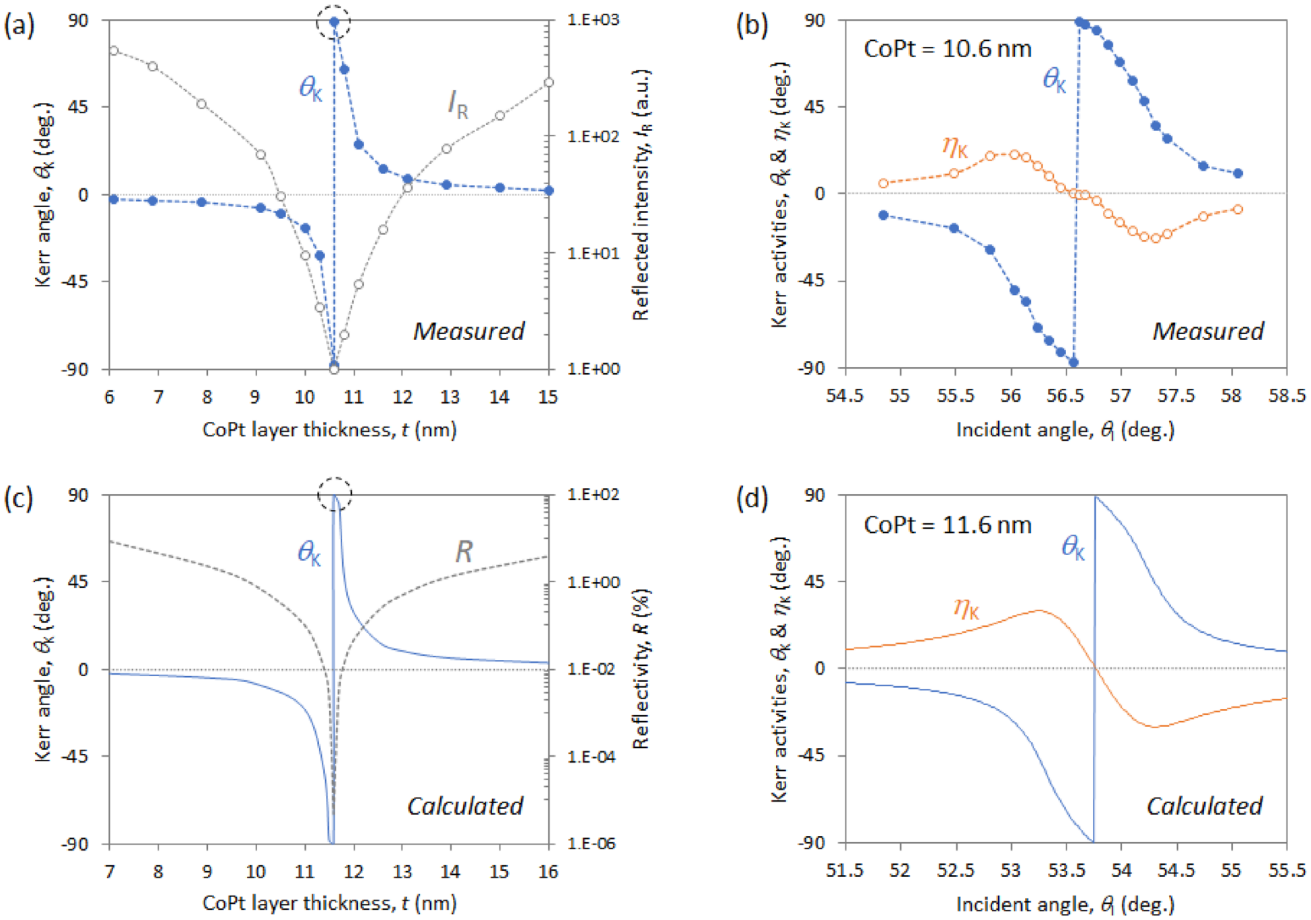
Experimentally measured (**a**,**b**) and analytically calculated (**c**,**d**) optical and MO responses for Al_2_O_3_ (5 nm)/CoPt (*t* nm)/AZO (30 nm) trilayers in the polar Kerr excitation geometry. (**b**) Measured and (**d**) calculated Kerr activities (*θ*_K_ and *θ*_K_) as a function of incident angle of radiation for samples containing 10.6- and 11.6-nm-thick CoPt layers, respectively. For the simulations at 658 nm, Table 1 lists the dielectric constants used for each layer.

The excitation of SPPs considerably affects the optical and MO responses of the CoPt trilayered films. Figure 2a shows the *I*_R_ and *θ*_K_ of the Al_2_O_3_ (5 nm)/CoPt (*t* = 6‒15 nm)/AZO (30 nm) trilayers in the saturated magnetic state and of varying CoPt thickness, in which the incident angle *θ*_I_ is optimized to obtain the largest *θ*_K_ in each sample. The *θ*_K_ is significantly enhanced for a CoPt thickness of 10.6 nm, where *I*_R_ is minimal due to SPR. This sample produces an extraordinary *θ*_K_ =  ± 88.9°, which is the almost upper limit of the rotation angle (± 90°) of the polarization, and the sign of *θ*_K_ is reversed under the SPR condition. The enhancement factor is approximately 300 compared with *θ*_K_ = 0.29° which is obtained in a common polar Kerr measurement (see “Methods” section).

Figure 2b shows how the MO activities depend on the incident angle *θ*_I_ for the 10.6-nm-thick CoPt sample. The sign of MO both parameters (*θ*_K_ and *η*_K_) is reversed at a resonance angle of 56.6°. The CoPt sample almost reaches the upper limit of *θ*_K_ =  ± 90° and *η*_K_ = 0° under the resonance conditions, which implies that the SPRs produce an orthogonal transformation of the polarization, transforming the *p*-polarized incident light to *s*-polarized light upon reflection. Moreover, the phase of the *s*-polarized reflected light reverses depending on whether the magnetization of CoPt is up or down.

Recently, we experimentally demonstrated the reversal of MO polarity and a large *θ*_K_ of ± 21° in a polar MO‒SPR element comprising CoPt/Ag bilayers^28^. However, the upper limit of Kerr rotation (± 90°) is only possible in the Al_2_O_3_/CoPt/AZO trilayers. Based on theory, Kaihara et al. claim that Al_2_O_3_/SiO_2_/Fe trilayers should display orthogonal transformation (90° rotation) and nearly-full-orbed deformation (44° ellipticity) in the polar Kerr geometry in the ATR configuration^33^. Their trilayered structure, comprising a ferromagnetic metal and two dielectric layers of low and high refractive indices, is same as the Otto configuration. Strong MO enhancement occurs at the cutoff condition of the waveguide modes, so it depends significantly on the thicknesses of two dielectric layers. By contrast, the proposed MO‒SPR element consisting of Al_2_O_3_/CoPt/AZO trilayers is in the Kretschmann configuration. The MO enhancement originates from the excitation of SPPs on the CoPt layer. The Al_2_O_3_ and AZO dielectric layers serve only as protective and seed layers, respectively, and basically do not affect the SPRs. As an example, the calculated reflectance and transmittance of the *p*-polarized light are provided in Fig. S1 (Supplementary Material). In the following theoretical calculations, CoPt single-layered samples without Al_2_O_3_ and AZO layers show similar MO spectra with the upper limit of Kerr rotation. Moreover, we experimentally realized the orthogonal transformation without using waveguide modes.

We now analyze theoretically the optical and MO responses of the Al_2_O_3_/CoPt/AZO trilayers. If the magnetization of magnetic films is aligned perpendicular to the film surface (along the *z* axis), which corresponds to the so-called polar Kerr configuration, the dielectric permittivity can be described with the dielectric tensor

1 $$\left[\epsilon \right]=\left[\begin{array}{ccc}{\varepsilon }_{xx}& i{\varepsilon }_{xy}& 0\\ -i{\varepsilon }_{xy}& {\varepsilon }_{xx}& 0\\ 0& 0& {\varepsilon }_{xx}\end{array}\right].$$

The optical and MO spectra can be calculated by an algorithm based on the 2 × 2 matrices method considering the dielectric tensor^34^. The present simulations use the actual dielectric permittivity obtained from the ellipsometric and polar Kerr spectra of each layer. The real and imaginary parts of the diagonal elements (*ε*_xx_ = *ε*_xx_′ + *iε*_xx_″) were determined from the optical constants (*N* = *n* + *ik*), which were measured with a spectroscopic ellipsometer. The real and imaginary parts of the off-diagonal elements (*ε*_xy_ = *ε*_xy_′ + *iε*_xy_″) were calculated on the basis of the complex polar Kerr activities (*Φ*_K_ = *θ*_K_ + *iη*_K_) and the diagonal elements^35^. The dielectric permittivities of each layer used for numerical simulations are described in Table 1 (see “Methods” section).

**Table 1:** Simulated dielectric constants of each layer at a wavelength of 658 nm.

Material	*ε*_xx_	*ε*_xy_
Al_2_O_3_	2.708 + 0.002*i*	‒
CoPt	− 12.952 + 21.368*i*	0.666–0.033*i*
AZO	3.947 + 0.020*i*	‒

The theoretical calculations are qualitatively consistent with the experimental results. Figure 2c shows the calculated reflectivity *R* and Kerr angle *θ*_K_ for the Al_2_O_3_ (5 nm)/CoPt (*t* nm)/AZO (30 nm) trilayers of varying CoPt thickness. The incident angle of irradiation was selected to maximize *θ*_K_ in each sample, and the AZO and Al_2_O_3_ layers were 30- and 5-nm thick, respectively, which are the same as the experimental samples. The calculated *θ*_K_ spectrum (blue solid curve in Fig. 2c) indicates a drastic response to the CoPt thickness, like the experimental results shown in Fig. 2a. The MO‒SPR element consisting of an 11.6-nm-thick CoPt layer produces the upper limit for *θ*_K_ (± 90° rotation), where *R* is minimal.

Figure 2d shows the MO spectra as a function of *θ*_I_ for this sample. The signs of *θ*_K_ and *η*_K_ are reversed at the resonance condition *θ*_I_ = 53.7°. These calculated optical and MO responses are qualitatively consistent with the experimental results, but the optimized CoPt thickness and the resonance angle differ slightly from the experimental values. One reason for these differences between theory and experiment may be due to atomic diffusion and roughness at the interfaces, due in particular to the oxidation of CoPt. We recently investigated how interfacial conditions affect the perpendicular magnetic anisotropy in CoPt/AZO stacked films^36^. The electronic hybridization between O 2*p* and Co 3*d* orbits across the interface improves the perpendicular magnetic properties caused by the increase in the magnetic surface anisotropy^36–38^.

### Magneto-reflective response

**Figure 3:**
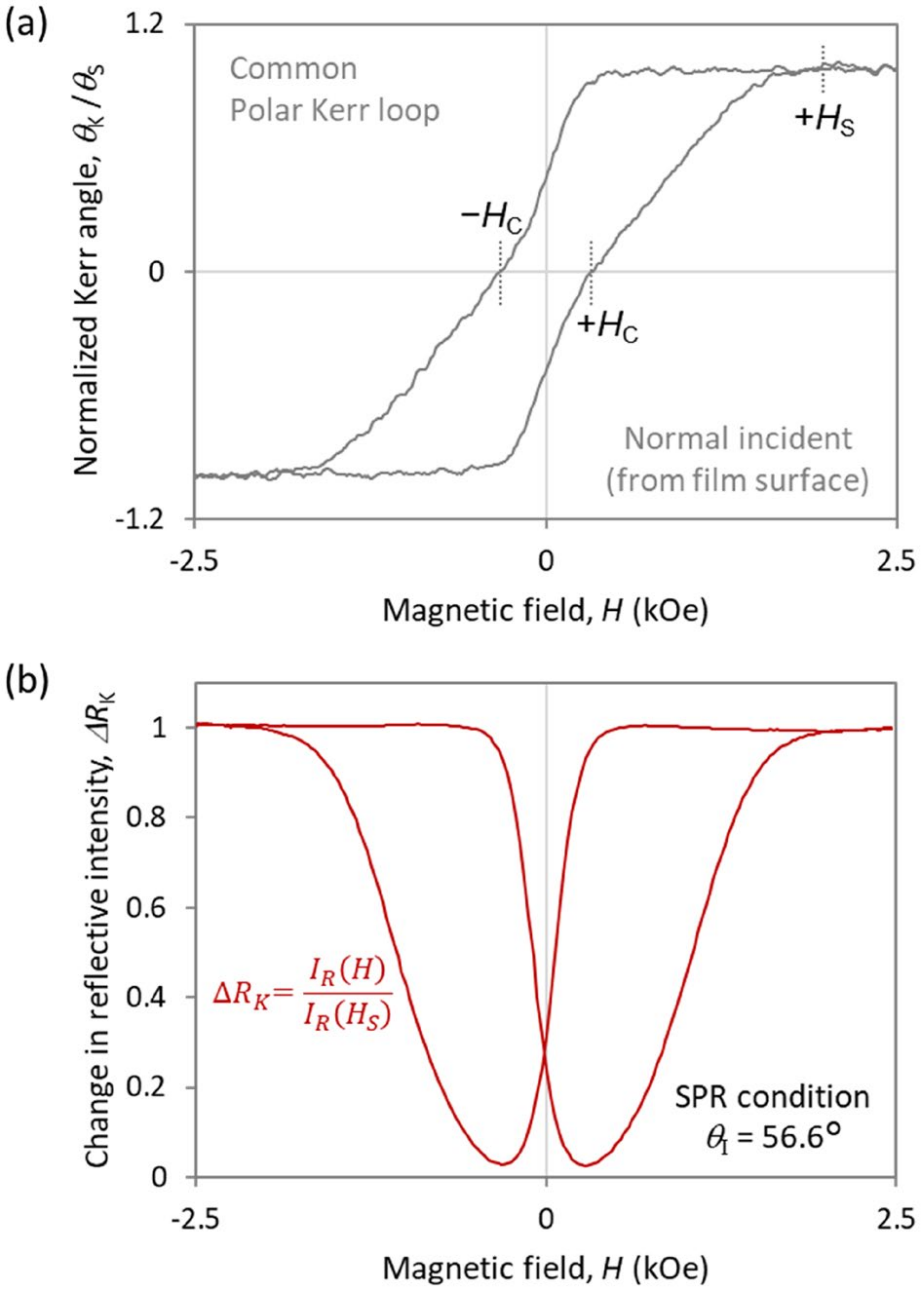
(**a**) MO hysteresis loop and (**b**) reflected intensity as a function of external magnetic field for Al_2_O_3_/CoPt/AZO trilayer shown in Fig. 2. (**b**). Both loops are normalized by each value in the magnetically saturated state. The MO loop was measured under the common polar Kerr configuration in which the incident radiation is normal to the sample surface. The change in reflective intensity was measured under SPR conditions.

Besides the MO activities, the reflected intensity (*I*_R_) of the CoPt trilayers depends substantially on the external magnetic field. Figure 3a,b shows the MO hysteresis loop in the common polar Kerr measurement and the change *ΔR*_K_ in reflected intensity under the SPR geometry, respectively. This MO‒SPR sample comprising a 10.6-nm CoPt, as shown in Fig. 2b, produces the polarization transformation (*θ*_K_ ≈ ± 90°). *ΔR*_K_ is normalized by the reflected intensity at the magnetic saturated state *H*_S_:

2 $${\Delta R}_{K}=\frac{{I}_{R}\left(H\right)}{{I}_{R}\left({H}_{S}\right)}.$$

The common polar Kerr measurement indicates a typical perpendicular magnetic hysteresis loop with a coercivity of *H*_C_ = 0.4 kOe and a magnetic saturation field of *H*_S_ = 2.0 kOe. Conversely, as shown in Fig. 3b, the application of the external magnetic field provokes a drastic response in the reflected intensity. The reflected intensity becomes minimum value at the coercivity (± *H*_C_) and increases significantly with increasing magnetic field until becoming constant above ± *H*_S_. Notice that the reflective intensity is almost zero in the demagnetized state (± *H*_C_).

This reflective response produced by the external magnetic field cannot be explained by the usual MO phenomena. For example, in the polar Kerr geometry, the reflectivity and the magnetization may be related by magnetic circular dichroism, which originates from the different absorption of left and right circularly polarized light in magnetic materials oriented parallel to the direction of light propagation. For linear polarized light containing left and right circularly polarized light, the reflected intensity basically decreases with increasing magnetization. By contrast, Fig. 3b shows that the reflected intensity of our sample increases with increasing magnetization of CoPt. Additionally, the magnitude of the magnetic circular dichroism is generally determined by *η*_K_, but, as shown in Fig. 2b, the CoPt sample has *η*_K_ = 0 under SPR conditions. Therefore, the change in the reflected intensity due to the external magnetic field does not originate from magnetic circular dichroism.

Another possible physical origin is that the modulation of the permittivity described by the dielectric tensor changes the reflectivity. For in-plane magnetic films in the transverse Kerr geometry, the magnetization reversal of the off-diagonal elements of the dielectric tensor changes the reflected intensity. As described in the introduction, the excitation of SPPs can significantly enhance this MO phenomenon. However, the response depends linearly on the magnetization, which differs from the quadratic response shown in Fig. 3b. Furthermore, the magnetic field can act on the diagonal elements of the dielectric tensor of magnetic multilayers. This MO phenomenon, known as the magnetorefractive effect^39^, is quadratic in the magnetization, and its origin is related to variations in the electric conductivity induced by the magnetic alignment in the multilayers. The magnetorefractive effect is usually detected in the mid- and far-infrared ranges because the conduction electrons can affect the optical response at these wavelengths, and it is small in the visible range. Consequently, the variation in reflective intensity of the CoPt samples due to the external magnetic field is not explained by these usual MO phenomena.

## Discussion

**Figure 4:**
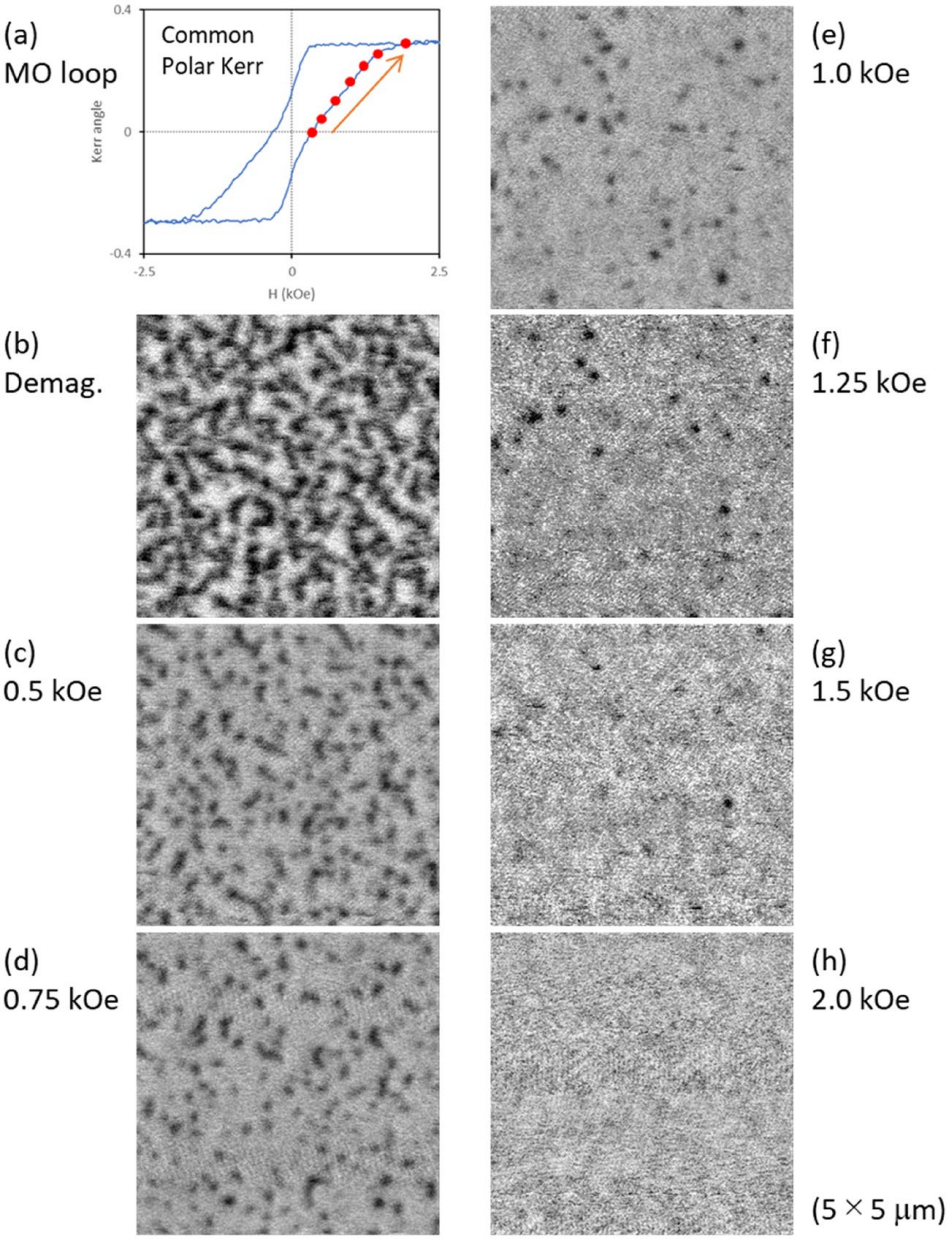
Change in magnetic domain structures due to an applied external magnetic field for Al_2_O_3_/CoPt/AZO trilayer shown in Fig. 3. (**a**) Common polar Kerr loop and (**b**–**h**) MFM images under external magnetic fields of 0 (after DC demagnetization), 0.5, 0.75, 1.0, 1.25, 1.5, and 2.0 kOe, respectively. Red closed circles in (**a**) show each MFM measurement state.

We propose that the magneto-reflective response in the MO‒SPR elements is related to the magnetic domain structure. To elucidate this, the magnetic domain of the trilayer consisting of a 10.6-nm CoPt was probed by magnetic force microscopy (MFM). Figure 4a–h shows the MO hysteresis loop of the common polar Kerr measurement and MFM images under external magnetic fields, respectively. Red closed circles in Fig. 4a indicate each MFM measurement state. The topographic image of the sample obtained simultaneously by atomic force microscopy indicated a surface roughness *R*_a_ = 0.18 nm with no identifiable features. Conversely, as shown in Fig. 4b, the MFM image at a dc-demagnetized state (after applying a magnetic field of *H*_C_ = 0.4 kOe) displays a maze-like domain pattern with high magnetic phase contrast. The presence of strong contrast in the MFM image suggests the development of perpendicular magnetic anisotropy. The magnetic domain structure gradually disappears as the external magnetic field increases, and the MFM image becomes flat (single color) when the magnetic field is saturated (Fig. 4h: *H*_S_ = 2.0 kOe).

**Figure 5:**
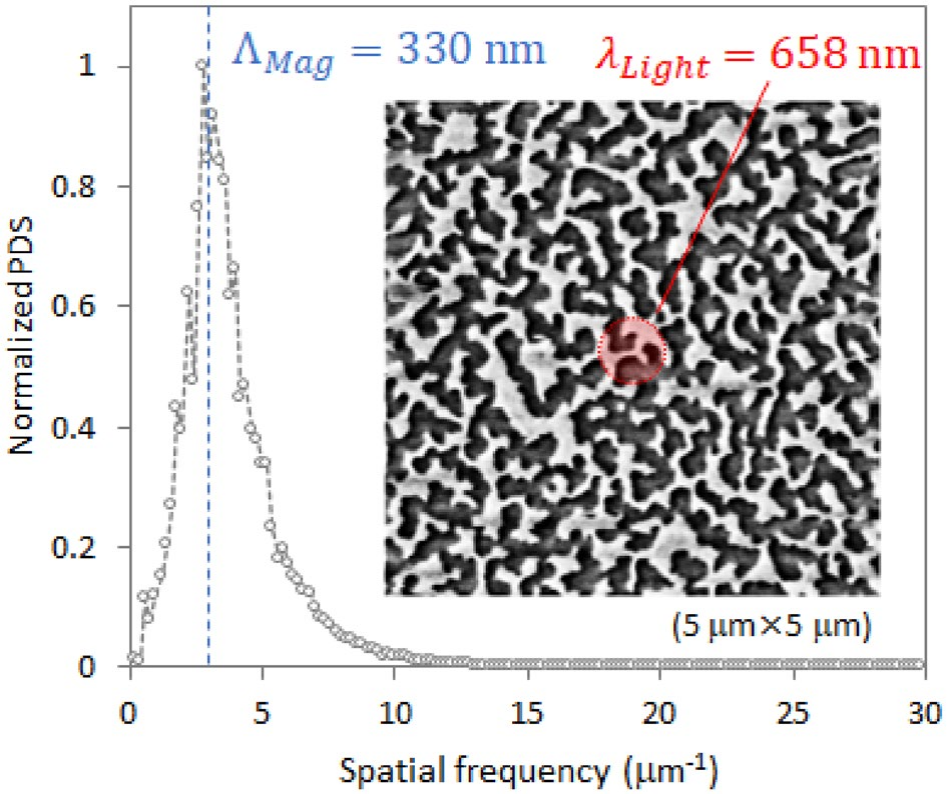
2D-PSD plot calculated from the 2D fast Fourier transform of the MFM image of the MO–SPR sample in the dc-demagnetized state. The red circle shows the size of the wavelength of the measurement light.

Figure 5 shows the two-dimensional (2D) isotropic power spectral density (PSD) calculated from the 2D fast Fourier transform of the MFM image at the demagnetized state. This profile provides the period distribution of the magnetic domains (i.e., twice the domain size) and their relative weight. Twice the mean size of the magnetic domain (*Λ*_mag_), obtained from the inverse of the spatial frequency at the peak of the PSD profile, is around 330 nm. The red circle in the MFM image shows the size of measurement wavelength. In the demagnetized state, the CoPt film comprises magnetic domains smaller than the wavelength of the incident radiation (*λ*_Light_ = 658 nm). Alternatively, the demagnetized CoPt film can be thought as a nonmagnetic material optically.

In general, when a *p*-polarized electric field (*E*_P_^i^) from a nonmagnetic medium incidents on a magnetic medium, in terms of the Fresnel reflection matrix, *M*, the reflected electric field (*E*_P_^r^, *E*_S_^r^) from the sample can be expressed in matrix notation as follows:

3 $$\left(\begin{array}{c}{E}_{P}^{r}\\ {E}_{S}^{r}\end{array}\right)=M\left(\begin{array}{c}{E}_{P}^{i}\\ {E}_{S}^{i}\end{array}\right)=\left(\begin{array}{cc}{r}_{pp}& {r}_{ps}\\ {r}_{sp}& {r}_{ss}\end{array}\right)\left(\begin{array}{c}{E}_{P}^{i}\\ 0\end{array}\right),$$

where *r*_*ij*_ is the ratio of the reflected *i*-polarized electric field to the incident *j*-polarized electric field, and expressed by the following equations in a polar Kerr geometry^40–42^:

4 $${r}_{pp}=\frac{{n}_{1}\mathrm{cos}{\theta }_{0}-{n}_{0}\mathrm{cos}{\theta }_{1}}{{n}_{1}\mathrm{cos}{\theta }_{0}+{n}_{0}\mathrm{cos}{\theta }_{1}},$$

5 $${r}_{sp}=\frac{i{n}_{0}{n}_{1}\mathrm{cos}{\theta }_{0}}{\left({n}_{1}\mathrm{cos}{\theta }_{0}+{n}_{0}\mathrm{cos}{\theta }_{1}\right)\left({n}_{0}\mathrm{cos}{\theta }_{0}+{n}_{1}\mathrm{cos}{\theta }_{1}\right)}\times \frac{{\varepsilon }_{xy}}{{\varepsilon }_{xx}}.$$

In the above equations, *θ*_0_ is the angle of incidence, and *n*_0_ and *n*_1_ are refractive indices of the nonmagnetic medium and magnetic medium, respectively. The complex refractive angle, *θ*_1_, of the magnetic medium is determined using Snell’s law. The complex Kerr activities can be defined as follows:

6 $${\Phi }_{K}={\theta }_{K}+i{\eta }_{K}={\mathrm{tan}}^{-1}\left(\frac{{r}_{sp}}{{r}_{pp}}\right).$$

The polarization transformation (*θ*_K_ ≈ ± 90°) shown in Fig. 2 is attributed to almost perfect absorption of the *p*-polarized component due to SPRs while a little amount of *s*-polarized remains (Supplementary Fig. S2).

The Fresnel coefficient *r*_pp_ does not depend on the magnetization, and the MO component *r*_sp_ equals zero when the magnetic medium is a demagnetized state (*ε*_xy_ = 0). Therefore, the ratio of reflectivities in demagnetized (*R*_demag_) and magnetized (*R*_mag_) states is calculated via

7 $$\frac{{R}_{demag}}{{R}_{mag}}=\frac{{\left|{r}_{pp}\right|}^{2}}{{\left|{r}_{pp}\right|}^{2}+{\left|{r}_{sp}\right|}^{2}}.$$

**Figure 6:**
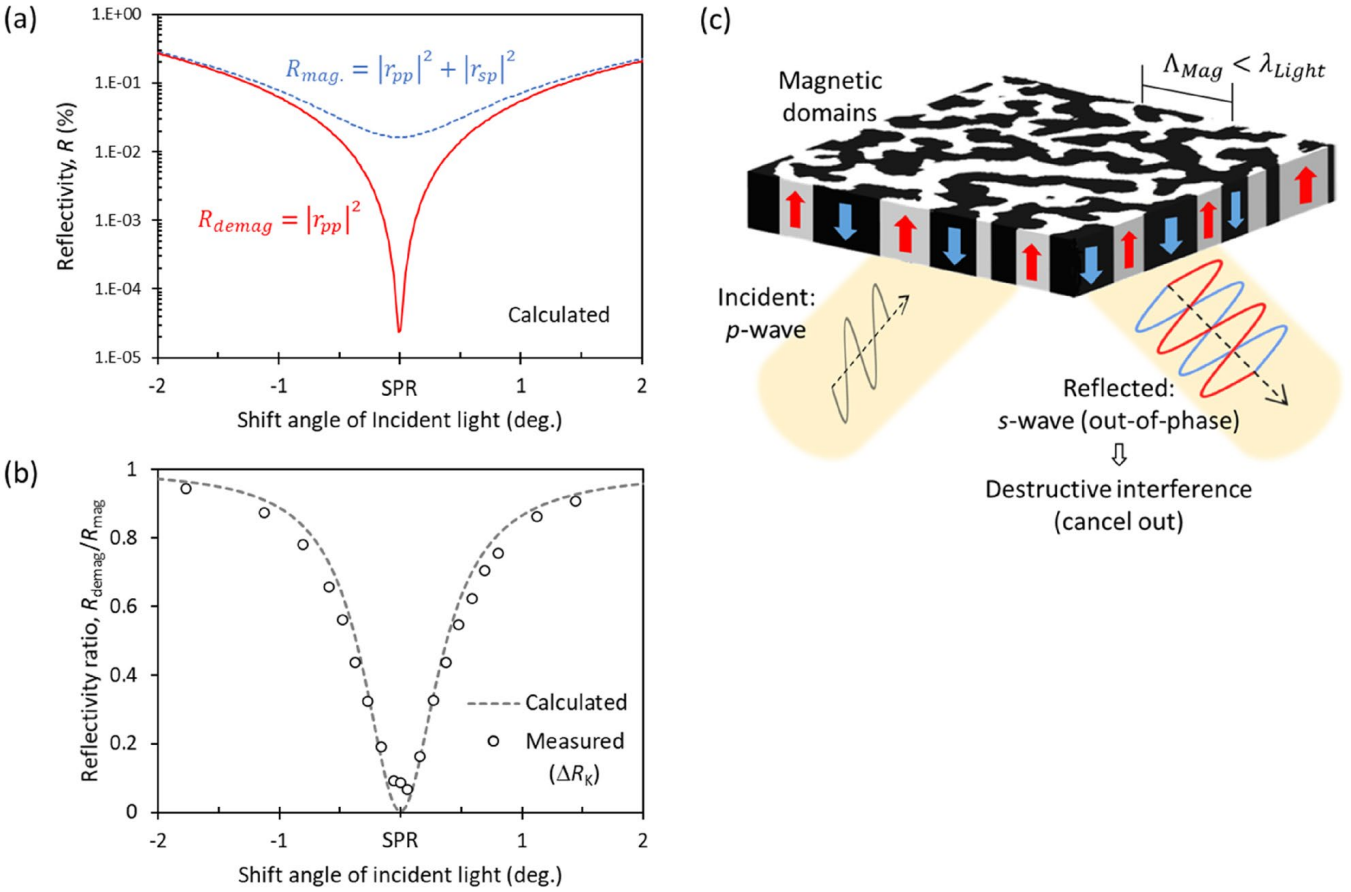
(**a**) Calculated reflective spectra for the MO–SPR element under magnetized (blue dashed line) and demagnetized (red solid line) states. (**b**) Calculated (gray dashed line) and measured (open circles) reflective ratios under both states. The reflective responses are plotted as a function of the shift angle from the SPR condition. (**c**) Schematic of MO destructive interference caused by the orthogonal transformation of polarization by subwavelength magnetic domain structure.

Figure 6a,b show the reflective spectra as a function of the shift angle of incident radiation from the SPR condition. In the calculations, Eqs. (4) and (5) were modified for the trilayered structure. For the *n*-th layer (boundary) in a multilayer, Eq. (3) is changed to the following equation:

8 $${\left(\begin{array}{c}{E}_{P}^{r}\\ {E}_{S}^{r}\end{array}\right)}_{n-1}={M}_{n}{\left(\begin{array}{c}{E}_{P}^{i}\\ {E}_{S}^{i}\end{array}\right)}_{n}.$$

We can calculate *M*_n_ interactively, starting at the surface (air) and moving down the multilayer until the substrate (prism). The subscriptions in Eqs. (4) and (5) are changed to the two media making the boundary. The blue dashed and red solid curves in Fig. 6a show the calculated reflectivities in the magnetized and demagnetized states, respectively. For both states under the SPR condition, *r*_pp_ is small (almost zero) due to the excitation of surface plasmons. However, in the magnetized state, *r*_sp_ is nonnegligible because the *s*-polarized component generated by the MO effect does not contribute to plasmon excitation. Thus, the magnetization under the SPR condition affects the reflectivity. The gray dashed curve in Fig. 6b shows the ratio of the demagnetized and magnetized reflectivities calculated using Eq. (7). The ratio of reflected intensities (*ΔR*_K_: open circles), which were measured at the *H*_C_ and *H*_S_, is also plotted in Fig. 6b. The experimental results are consistent with the theoretical calculations.

This magneto-reflective response can be also explained by an MO interference caused by the subwavelength magnetic domain structure. Figure 6c shows a schematic of the interference phenomenon on an MO‒SPR element consisting of perpendicular magnetic film with *θ*_K_ =  ± 90°. As discussed above, excitation of SPPs transforms the incident *p*-polarized radiation into reflected *s*-polarized radiation. The reflected *s*-polarized waves are out of phase according to the magnetic direction. In the demagnetized state, the magnetic domains are smaller than the wavelength, and the magnetic up and down domains have the same surface area. This is the same situation as an antireflective coating implemented in common metasurfaces such as 2D gratings with optimized height and fill factor. Artificially subwavelength structured surfaces, called metasurfaces, reduce reflections^43–45^. The effective refractive index is arranged by the height and fill factor of the subwavelength patterns. For example, when the optical height of 2D gratings equals a quarter wavelength of the incident radiation, the optimized fill factor causes destructive interference of normally incident radiation. If the same intensity is reflected from the grating and substrate surfaces, they cancel each other because they are out of phase. In the demagnetized state comprising the subwavelength magnetic domains, two waves reflected from magnetic up and down domains cancel out because they have the same intensity and are out of phase. The reflectivity in the demagnetized state goes to zero due to the destructive interference. This MO interference phenomenon is quadratic in applied magnetization because one area of the magnetic domains increases with increasing magnetization, which is consistent with the results shown in Fig. 3a,b.

In recent years, metasurfaces with subwavelength structured surfaces have become an emerging research area because of their ability to control the polarization, phase, amplitude, and dispersion of light^46,47^. Common metasurfaces usually have difficulty modulating the optical functions after device fabrication because doing so would require modifying the structural features such as height, direction, and fill factor. Conversely, with subwavelength magnetic domain structures, the fill factor is easily modified by applying an external magnetic field. The MO‒SPR system with the functionality of polarization transformation may be of use in such optically active devices.

## Conclusion

To summarize, we show experimentally that, in the ATR (Kretschmann) configuration, the MO‒SPR elements comprising CoPt perpendicular magnetic films almost produce the upper limit of Kerr rotation (*θ*_K_ =  ± 88.9°). *P*-polarized radiation incident on the CoPt film is reflected as *s*-polarized radiation that is out of phase depending on whether the magnetization is up or down. These magneto-plasmonic phenomena are also described by theoretical calculations. Moreover, we demonstrate the drastic response of the reflected intensity induced by the application of an external magnetic field. The reflectivity goes almost to zero in the demagnetized state and increases with increasing magnetization. This MO phenomenon is attributed to a destructive interference caused by the subwavelength magnetic domain structures that orthogonally transform the incident radiation and reverse its phase depending on the magnetic direction. Such magneto-plasmonic systems with subwavelength magnetic domains may be of use in optically active devices.

## Methods

Samples comprising Al_2_O_3_ (5 nm)/CoPt (*t* = 6–15 nm)/AZO (30 nm) trilayers were fabricated via magnetron sputtering onto a glass substrate at ambient temperature. CoPt layers with an hcp–(001) orientation of the surface normal were deposited from a composite target of Co[80]–Pt[20] (at.%). The AZO underlayer was deposited from a ZnO target containing dilute Al_2_O_3_ at 0.1 wt%, and the *c*-plane (001) faced wurtzite structure improved the perpendicular magnetic properties of CoPt. Out-of-plane and in-plane X-ray diffraction measurements revealed that the AZO underlayer increased the crystalline orientation and reduced atomic stacking faults in the hcp–(001) CoPt films, increasing the perpendicular magnetic anisotropy of CoPt. The magnetic properties and crystalline structure of the CoPt/AZO stacked films are detailed in Ref.^36^.

The fundamental MO properties (*θ*_K_ and *η*_K_ spectra) of the samples were measured using a common polar Kerr spectroscopy with a photoelastic modulator. In the common polar Kerr system, linearly polarized radiation was emitted from a monochromatic Xe light source to irradiate the sample surface along the surface normal. The wavelength range and resolution were 250–900 nm and 1.0 nm, respectively, and a maximum magnetic field of ± 25 kOe was applied normal to the sample surface. The surface topography and magnetic domains of the samples were observed via MFM. The MFM images under external magnetic fields were obtained in a vacuum.

The performed numerical simulations were based on a 2 × 2 matrices algorithm^34^. The dielectric constants obtained from the ellipsometric and polar Kerr spectra of the single films were used in the simulations. The real and imaginary parts of the diagonal elements were determined from the optical constants, which were measured using a spectroscopic ellipsometer. The real and imaginary parts of the off-diagonal elements of CoPt were calculated from the diagonal elements and fundamental *θ*_K_ and *η*_K_ values^35^, which were determined using a 100-nm-thick CoPt single film in common polar Kerr measurements. Table 1 lists the dielectric constants of each layer in the simulations for a 658-nm wavelength.

## Supplementary Information

Supplementary Figures.

## Data Availability

The datasets generated and/or analyzed during the current study are available from the corresponding author on reasonable request.
