# Supplementary material for: Polarization transformation and destructive interference on subwavelength magnetic domains in magneto-plasmonic systems
Source: Sci Rep. 2022 Aug 16;12:13871. doi: 10.1038/s41598-022-17971-w (PMC9381552; doi:10.1038/s41598-022-17971-w)
Supplement: Supplementary file 1 — Supplementary Figures. [file 41598_2022_17971_MOESM1_ESM.pdf]

Supplementary

Polarization transformation and destructive interference on subwavelength magnetic domains in magneto-plasmonic systems

Haruki Yamane*, Satoshi Yanase, Takashi Hasegawa, Masanobu Kobayashi, Yukiko Yasukawa, *yamane@aitc.pref.akita.jp

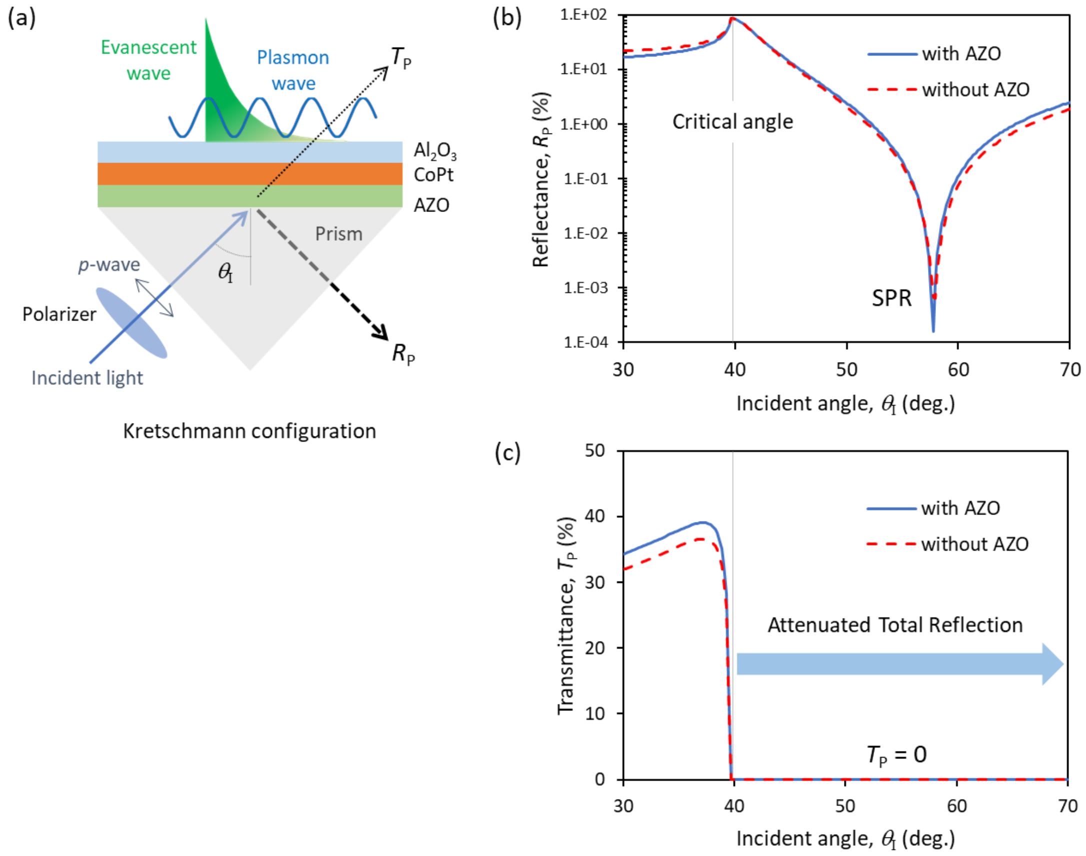

Figure S1. (a) Schematic of experimental setup for the Kretschmann (or attenuated total reflection) configuration. Calculated (b) reflectance R_P and (c) transmittance T_P of *p*-polarized light for CoPt stacked films with and without a 30-nm AZO underlayer. Bule solid and red dashed lines represent [Al_2O_3 (5 nm)/CoPt (11.6 nm)/AZO (30 nm)] and [Al_2O_3 (5 nm)/CoPt (11.5 nm)], respectively.

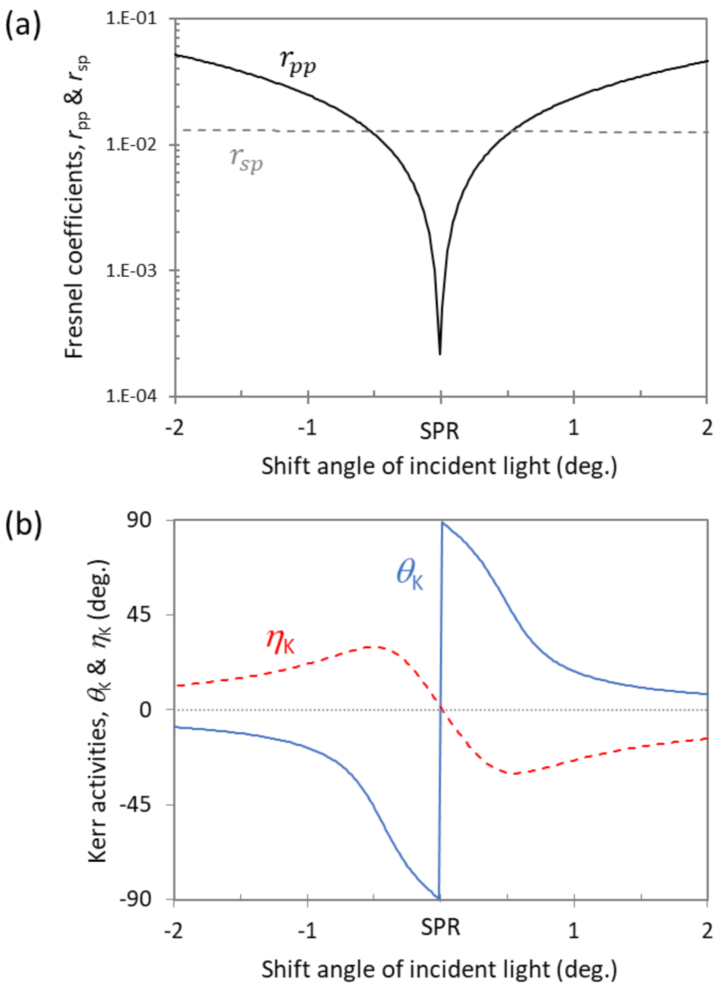

Figure S2. Calculated spectra of (a) Fresnel coefficients r_{pp} (black solid line) and r_{sp} (gray dashed line), and (b) polar Kerr activities θ_K (blue solid line) and η_K (red dashed line) for [Al_2O_3 (5 nm)/CoPt (11.6 nm)/AZO (30 nm)]. For these calculations, we used Eqs. (4) and (5) by modifying them for the trilayered structure. The optical and MO responses are plotted as a function of the shift angle from the SPR condition.
